# Entrepreneurial tendency of Nursing students: a comparison between graduating beginners and undergraduate students*

**DOI:** 10.1590/1518-8345.4397.3402

**Published:** 2021-01-08

**Authors:** Liana Amorim Corrêa Trotte, José Luís Guedes dos Santos, Caroline Ferreira Neris Sarat, Maria Gefé da Rosa Mesquita, Marluci Andrade Conceição Stipp, Patrícia de Souza, Quézia Guedes de Mello Duarte, Bruno de Campos Gobato, Claudia Feio da Maia Lima

**Affiliations:** 1 Universidade Federal do Rio de Janeiro, Escola de Enfermagem Anna Nery, Rio de Janeiro, RJ, Brazil.; 2 Universidade Federal de Santa Catarina, Departamento de Enfermagem, Florianópolis, SC, Brazil.; 3 Universidade Federal de Mato Grosso do Sul, Departamento de Enfermagem, Campo Grande, MS, Brazil.; 4 Centro Universitário Augusto Motta, Departamento de Enfermagem, Rio de Janeiro, RJ, Brazil.; 5 Universidade Federal de Santa Catarina, Florianópolis, SC, Brazil.; 6 Scholarship holder at the Programa Institucional de Bolsas de Iniciação Científica (PIBIC) / Conselho Nacional de Desenvolvimento Científico (CNPq), Brazil.; 7 Universidade Federal do Recôncavo da Bahia, Centro de Ciências da Saúde, Santo Antônio de Jesus, BA, Brazil.; 8 Scholarship holder at the Fundação de Amparo à Pesquisa do Estado da Bahia (FAPESB), Brazil.

**Keywords:** Job Market, Entrepreneurship, Students, Nursing, Education, Nursing, Nurse’s Role, Nursing Administration Research, Mercado de Trabalho, Contrato de Risco, Estudantes de Enfermagem, Educação em Enfermagem, Papel do Profissional de Enfermagem, Pesquisa em Administração de Enfermagem, Mercado de Trabajo, Acuerdo de Riesgo, Estudiantes de Enfermería, Educación em Enfermería, Rol de la Enfermera, Investigación en Administración de Enfermería

## Abstract

**Objective:**

to compare the entrepreneurial tendency between beginner and graduating students from undergraduate Nursing courses.

**Method:**

this is a cross-sectional and quantitative research study. Data was collected from 377 Nursing students from four undergraduate Nursing courses in different Brazilian regions, 162 of them in first year and 215 in last year. Data was collected by means of a social and academic characterization form and the General Entrepreneurial Tendency Test. Data analysis was conducted by means of descriptive and inferential statistics.

**Results:**

the scores of the beginner students were below the mean in all dimensions of the instrument. The senior year students were above the test mean in the *Impulse and determination* dimension. A statistically significant difference was identified in relation to the course period and to the entrepreneurial tendency in the following dimensions: *Need for achievement* (p=0.001) and *Impulse and determination* (p=0.000).

**Conclusion:**

the results indicate the importance of investment by universities in the development of an entrepreneurial culture in higher education in Nursing.

## Abstract (pt)

**Objetivo:**

comparar a tendência empreendedora entre alunos iniciantes e concluintes de cursos de graduação em enfermagem.

**Método:**

trata-se de uma pesquisa quantitativa, de caráter transversal. Os dados foram coletados com 377 estudantes de enfermagem de quatro cursos de graduação em enfermagem de diferentes regiões do Brasil, sendo 162 do primeiro ano e 215 do último ano. Coletaram-se os dados por meio de uma ficha de caracterização social e acadêmica e o Teste de Tendência Empreendedora Geral. A análise dos dados foi meio de estatística descritiva e inferencial.

**Resultados:**

as pontuações dos alunos iniciantes ficaram abaixo da média em todas dimensões do instrumento. Os alunos concluintes ficaram acima da média do teste na dimensão *Impulso e determinação*. Identificou-se diferença estatisticamente significativa em relação ao período do curso e a tendência empreendedora nas dimensões: *Necessidade de realização* (p=0,001) e *Impulso e determinação* (p=0,000).

**Conclusão:**

os resultados indicam a importância do investimento das universidades no desenvolvimento de uma cultura empreendedora no ensino superior em enfermagem.

## Abstract (es)

**Objetivo:**

comparar la tendencia emprendedora entre estudiantes de primer año y concluyentes de la carrera de Enfermería.

**Método:**

se trata de una investigación cuantitativa de carácter transversal. Se recolectaron datos con 377 estudiantes de Enfermería de cuatro cursos de grado en Enfermería en diferentes regiones de Brasil, 162 de ellos en primer año y 215 en el último año de la carrera. Los datos se recolectaron por medio de un formulario de caracterización social y académica y por medio de la Prueba de Tendencia Emprendedora General. El análisis de datos se realizó por medio de estadística descriptiva e inferencial.

**Resultados:**

las puntuaciones de los estudiantes de primer año estuvieron por debajo de la media en todas las dimensiones del instrumento. Los estudiantes concluyentes estuvieron por encima de la media de la prueba en la dimensión *Impulso y determinación*. Se identificó una diferencia estadísticamente significativa en relación con el período de la carrera y la tendencia emprendedora en las siguientes dimensiones: *Necesidad de realización* (p=0,001) e *Impulso y determinación* (p=0,000).

**Conclusión:**

los resultados indican la importancia de la inversión de las universidades en el desarrollo de una cultura emprendedora en la educación superior en Enfermería.

## Introduction 

In general, entrepreneurship is defined as the realization or introduction of something new and different from what is traditionally done, based on the identification of unmet opportunities or needs^(^ 1 ^)^. It can be developed in different forms, the three main ones being: business entrepreneurship, intra-entrepreneurship, and social entrepreneurship. The first typology is better known and consists of the creation of a company or business that allows for autonomous professional practice. In intra-entrepreneurship, the entrepreneurs are motivated by the growth of the company or organization with which they have an employment relationship, while social entrepreneurs want to promote social changes in the context in which they are inserted^(^ 1 ^-^ 2 ^)^.

These three forms of entrepreneurship apply to Nursing. The nurse can have a company to work directly with patient care or providing consultancy services. It also highlights its potential in the development of technologies and the search for innovations in care processes within the health services. In addition, it acts as a transformation agent and seeks better conditions for the care of individuals, families and communities in the health systems^(^ 2 ^-^ 3 ^)^.

In this sense, the importance of the nurses’ entrepreneurial potential in the operationalization of good health care practices, as well as for social and economic development, is discussed worldwide. This is due to the numerical representativeness of Nursing professionals as the largest workforce in the health area and their presence in the most diverse care scenarios, which can contribute and enhance the universal offer and coverage of health care in an integrated, continuous, and interdisciplinary way^(^ 4 ^-^ 5 ^)^.

For an entrepreneurial performance, specific skills are needed, such as, for example: communication, leadership, decision-making, and problem-solving skills^(^ 2 ^-^ 3 ^,^ 6 ^)^. Thus, it is important to develop and improve these skills throughout the Nursing training, by means of innovative teaching methodologies and strategies, and the existence of university management capable of influencing the formation of an entrepreneurial profile. It is essential to instigate in the student the knowledge of the different areas of the professional practice and how nurses can exercise their autonomy, in addition to stimulating their characteristics to act as future entrepreneurs in the profession. For the development of these skills and entrepreneurship in Nursing, courses and practices that guide creative and innovative activities should be included in the Nursing curriculum, as well as contributing to skills for coping with risks, and with undefined and complicated situations^(^ 7 ^)^.

In view of the increasingly dynamic and competitive contemporary work context, entrepreneurial characteristics are an important differential for the insertion and professional development of nurses in the health work market. In addition, an entrepreneurial behavior represents an opportunity to establish new relationships with the social context, act to guarantee comprehensive care, make decisions, and intervene in the work process with a focus on improving health care practices and the visibility of the profession^(^ 2 ^-^ 4 ^)^.

Worldwide, some universities have already adopted the premise that entrepreneurial education can contribute to a country’s social and economic development, such as the Massachusetts Institute of Technology and Stanford University that offer courses related to entrepreneurship. In this perspective, the university is considered as the place for the dissemination and deepening of this theme, because it is in the process of academic training that critical thinking, the formation of opinions and the dissemination of knowledge are built^(^ 3 ^,^ 8 ^)^.

However, even though it is an area of transversal knowledge for many professions, entrepreneurship is explained in a variable and uneven manner between higher education courses in Brazil. While in the Administration course, 65% of the students have already taken an entrepreneurship discipline, only 27% had an opportunity in the health sciences area^(^ 8 ^)^, which corroborates the need to raise awareness on the subject in the context of Nursing, mainly in the sense of knowing how this approach occurs throughout the training of nurses.

It is important to note that there is a gap in scientific knowledge related to entrepreneurship among Nursing students in Brazil. A search the Virtual Health Library (*Biblioteca Virtual da Saúde*, BVS), using the terms “nursing” and “entrepreneurship”, with no time frame, resulted in 36 articles. Considering the publications since 2010, 26 articles were found, but two studies were duplicated and three were editorials, thus totaling 21 articles. Of these studies, two^(^ 9 ^-^ 10 ^)^ were related to undergraduate Nursing education, but none of them addressed the entrepreneurial tendency of undergraduate Nursing students.

Similarly, two recent literature reviews on entrepreneurship in Nursing^(^ 2 ^,^ 3 ^)^, which included international databases such as Publisher Medline (PubMed), SciVerse Scopus (SCOPUS), Cumulative Index to Nursing and Allied Health Literature (CINAHL), and Education Resource Information Center (ERIC), also highlighted the shortage of studies on entrepreneurship in Nursing education. It is noteworthy that there were no studies comparing specifically the entrepreneurship of students beginning and finishing the Nursing course in the scientific production on the theme^(^ 3 ^,^ 7 ^,^ 11 ^)^. Thus, the relevance and contribution of this study to the national and international scientific community is justified.

From the above scenario and considering the approach to entrepreneurship throughout the education of nurses as an object of study, the following guiding question of the research was defined: Is there a difference between the entrepreneurial tendency of students who are beginning and finishing the undergraduate Nursing course?

Thus, this study aimed to compare the entrepreneurial tendency of students beginning and finishing the undergraduate Nursing course.

## Method

This is a cross-sectional and quantitative study, developed from a multicenter study in four Public Higher Education Institutions in different Brazilian regions: Southeast (Rio de Janeiro), South (Santa Catarina), Midwest (Mato Grosso do Sul), and Northeast (Bahia). The definition of the institutions was intentional, in order to consider the regional diversity and the professional contracts of the researchers.

The research scenarios were the four undergraduate Nursing courses of the universities participating in the study. Sampling was not probabilistic, as it predicted the application of the instruments to the total population of the study. The inclusion criteria used were as follows: being regularly enrolled in the Nursing course and attending the first or last period of the course. The exclusion criterion was the following: participants with active enrollment lockout at the time of data collection. Thus, the total number of research participants was 377 Nursing students, 162 (43%) from first year and 215 (57%) from last year.

Data collection took place in the first semester of 2018; the students were approached in the intervals of class hours on the *campi* of the universities. A three-part instrument was applied. The first contained questions about sociodemographic and academic aspects to characterize the sample (age, gender, marital status, technical course in Nursing, participation in a research or extension group, research scholarship, extension scholarship, and paid activity).

The second part of the instrument assessed the students’ perception of the relationship between entrepreneurship and Nursing, through three statements: 1) Entrepreneurship applies to Nursing; 2) The content of entrepreneurship is important in the training of nurses; and 3) Entrepreneurship is addressed during the undergraduate Nursing course. For each one, the respondents should indicate their agreement using a scale from zero to 10. The higher the value indicated, the greater the agreement.

In the third part of the questionnaire, there was the “General Entrepreneurial Tendency Test” (GET)^(^ 12 ^)^, which divides entrepreneurial characteristics into five dimensions: *Need for Success*, *Need for Autonomy/Independence*, *Creative Trend*, *Propensity to Calculated Risks* and *Impulse and Determination*. That scale was developed by Dr. Sally Caird in the Durham University Business School in 1990, has a behavioral focus and is increasingly used in Brazilian research studies^(^ 13 ^)^, including in the area of Nursing^(^ 3 ^,^ 11 ^,^ 14 ^)^.

The test consists of 54 statements, for which the respondent must mark “I agree” or “I disagree”. In odd questions, one point is added for each disagreement noted; in the even questions, a point for the indicated agreement is added. Thus, the score for each question is added and the final sum of each dimension is added. For the *Need for Achievement* dimension, the maximum GET score is 12 and the mean score is 9. In the *Need for Autonomy/Independence* dimension, the maximum score is 6 and the mean is 4. For the other dimensions, the maximum score is 12 and the mean is 8. Therefore, the entrepreneurial trend increases the higher the mean score in each dimension^(^ 12 ^)^.

For data analysis, the *Kolmogorov-Smirnov* normality test was applied, which showed that the data followed a normal distribution. Descriptive statistical tests and relative and absolute frequency tests were used. In the inferential analysis, the *Student* t test for independent samples was applied. The level of significance used as a parameter was 5%. The Statistical Package for the Social Sciences (SPSS) for Windows program, version 19.0, was used.

The study was approved by the Ethics and Research Committee, under CAEE number 66306117.9.2008.5238. All the national and international recommendations for research with human beings were followed.

## Results 

A total of 377 Nursing students participated in the study, 162 (43%) from first year and 215 (57%) from last year. The mean age of the students was 22.5 (±5.2) years old. The sociodemographic characterization of the study participants is shown in Table 1.

**Table 1:** Sociodemographic characterization of the Nursing students Florianópolis, SC, Brazil, 2018

Variables	N	%
Age		
up to 20 years old	134	35.5
21-22 years old	104	27.6
23-24 years old	64	17.0
25 years old or more	75	19.9
Gender		
Female	331	88.5
Male	43	11.5
Marital status		
Single	338	89.7
Married	22	5.8
Separated	1	0.3
Stable union	13	3.4
Others	3	0.9
Technical Course in Nursing		
Yes	36	9.5
No	341	90.5

Table 2 shows the academic and professional activities developed by the Nursing students. Graduating students have greater participation in research or extension groups and demonstrated greater engagement in their performance as scholarship holders and in the performance of paid professional activities.

**Table 2:** Academic and professional activities of the Nursing students. Florianópolis, SC, Brazil, 2018

Students	Beginners		Concluding	
Variables	N	%	N	%
Participation in research or extension group				
Yes	16	10%	154	71.6%
No	144	90%	61	28.4%
Research Scholarship				
Yes	2	1.2%	54	25.5%
No	155	96.3%	139	65.6%
Volunteer	4	2.5%	19	9.0%
Extension Scholarship				
Yes	5	3.1%	32	15%
No	157	98.1%	149	71%
Volunteer	2	1.2%	17	8.1%
Paid activity				
Yes	5	3.1%	32	15%
No	156	96.9%	182	85%

As for the relationship between entrepreneurship and Nursing, the mean values obtained indicate that the students consider that this theme is applicable and important for the profession and training of nurses. However, the view prevails that it is rarely addressed in undergraduate Nursing, especially among senior year students (Table 3).

**Table 3:** Relationship between entrepreneurship and Nursing. Florianópolis, SC, Brazil, 2018

Students	Beginners		Concluding		p-value^†^
Question	Mean	SD*	Mean	SD*	
Entrepreneurship applies to Nursing	8.19	1.99	8.77	1.91	0.441
The content of entrepreneurship is important in the training of nurses	8.11	2.15	8.98	2.36	0.000
Entrepreneurship is addressed during the undergraduate Nursing course	4.96	3.14	4.90	3.45	0.039

* SD = Standard deviation;

† *Student's* t test

Table 4 shows the behavior of the Nursing students in relation to the five entrepreneurial trends. The scores of the beginning students were below the mean in all the dimensions. The senior year students were above the mean of the test in the *Impulse and Determination* dimension, which is 8 points. A statistically significant difference was identified in relation to the course period and to the entrepreneurial tendency in the following dimensions: *Need for Achievement* (p=0.001) and *Impulse and Determination* (p=0.000). Beginning students obtained higher mean values than senior year students in the other four dimensions of the instrument.

**Table 4:** Entrepreneurial trend of beginning and concluding students. Florianópolis, SC, Brazil, 2018

Students	Beginners		Concluding		p-value†
Entrepreneurial trend	Mean	SD*	Mean	SD*	
Need for achievement	7.71	2.75	8.00	2.2	0.001
Need for autonomy/independence	3.67	1.73	3.39	1.68	0.920
Creative trend	6.65	2.03	6.33	1.97	0.885
Propensity to calculated risks	6.73	2	6.52	2.18	0.074
Impulse and determination	7.34	1.96	8.02	2.54	0.000

* SD = Standard deviation;

† *Student's* t test

## Discussion

The sociodemographic characterization of the participants in this study is similar to the profile of students in the health field from other federal universities in Brazil, in which young women predominate, aged between 18 and 24 years old and single^(^ 14 ^-^ 15 ^)^. In addition, the predominantly female profile of the participants portrays a historical characteristic of the Nursing profession in Brazil and in the world, which has been perpetuating over time^(^ 16 ^)^.

In relation to the academic and professional activities, the involvement of the Nursing students in research and extension activities was highlighted, especially by those in the final stretch of the course. Such a result was already expected, as most of the students in the early stages of the course have not yet had the opportunity to get in touch with these activities. In addition, this finding reinforces the prominence of public universities as the main institutions responsible for the development of research and extension in the country, as well as the incentive for student engagement in actions of this nature.

As for the relationship envisaged by the students between entrepreneurship and Nursing, it was identified that the perception of the approach to the theme throughout the training is lower than the importance they attach to the content for the nurse’s practice. Thus, the results suggest that the teaching of entrepreneurship is not a content widely addressed in the curriculum of undergraduate Nursing courses at the analyzed universities. However, this is not just a Brazilian problem. In the United States, most undergraduate Nursing courses do not teach concepts of innovative behavior and entrepreneurship, despite the recommendations of multidisciplinary and Nursing organizations that support the need for nurses to act as innovative agents and promoters of change^(^ 17 ^)^.

From the results of the GET, it was identified that the beginning students obtained scores below the mean in the five dimensions of the test. The graduating students were above the mean in only one of the dimensions of the instrument: *Impulse and Determination*.

This result can be explained by the lack of formal education on entrepreneurship throughout the undergraduate course, in order to develop specific skills and competences for an entrepreneurial profile^(^ 17 ^-^ 18 ^)^. Furthermore, knowledge on the subject is still embryonic in the country, which makes it difficult to disseminate the entrepreneurial culture in the university environment^(^ 19 ^-^ 20 ^)^.

The dissemination of an entrepreneurial culture in the academic environment requires discussions about the teachers’ competences in the health area. The results of a systematic review indicate that leadership and entrepreneurship are competencies assessed as being of average knowledge by the teachers of the area^(^ 21 ^)^. The existence of a faculty with an entrepreneurial spirit and that recognizes the need to educate the Nursing students about innovation is necessary to support education, research and professional development initiatives^(^ 17 ^,^ 19 ^)^.

The training institutions need to incorporate the perspective that entrepreneurship in Nursing enables different perspectives of performance, which differs from the traditional work patterns, providing opportunities for individual and economic development through the creation of new ways of acting in the labor market^(^ 22 ^-^ 23 ^)^. Along the same lines of thought, an Australian study also highlights the importance of supporting class bodies in defining specific policies for the development of entrepreneurship in the Nursing practice^(^ 24 ^)^.

In Brazil, studies using the GET in Nursing have been carried out previously and also showed that students and nurses had a low entrepreneurial tendency^(^ 14 ^,^ 18 ^,^ 25 ^)^. Nursing professionals have been widely absorbed by the hospital services, mainly by the Unified Health System (model adopted in Brazil) and by private services, which can lead nurses to seek work as employees, especially in countries like Brazil, with a still unstable economy^(^ 26 ^)^.

Another aspect to be signaled is the taboo that this theme represents to the profession. Historically, Nursing has been seen as a women’s work associated with charity and donation, with limited potential to be remunerated and offered freely through entrepreneurship. In addition, the vast majority of nurses work as salaried professionals, and the monetary exchange between professionals and patients^(^ 2 ^)^ is unusual and often considered unethical.

Despite the general results of the students’ entrepreneurial tendency being low, it is important to highlight that the graduating students presented mean values with a statistically significant difference in the following dimensions: *Need for Achievement* and *Impulse and Determination*. A high score on these items can indicate that future nurses, as they approach insertion in the job market, are more attentive in the search for opportunities or in creating them, with self-confidence and determination in their attitudes and knowledge to fulfill their professional dreams and objectives^(^ 27 ^)^.

In contrast, students in the initial period of the course do not have even greater clarity about the role of the nurse and the possibilities of the field of activity, which can explain the findings of the study. Entrepreneurship is associated with a feeling of seeking to find the world and bring new meanings to life, capable of promoting recognition and professional advancement^(^ 2 ^,^ 27 ^)^. Such feeling can intensify throughout the training process.

The nurse has a humanistic training that is guided by ethics, which allows looking at all the dimensions that make up the human being, always thinking about the integrality and quality of care. In this sense, social entrepreneurship stands out in Nursing and collaborates in the training of professionals who are agents of significant positive changes for patients and families^(^ 22 ^)^. Nurses know what needs to be developed in their communities, what services are needed or how they can be delivered more effectively, so as to achieve different health goals and objectives^(^ 5 ^)^.

In addition to social entrepreneurship, there is also business and intra-entrepreneurial in Nursing^(^ 2 ^,^ 3 ^,^ 28 ^)^. Thus, it can be considered that entrepreneurship is not only an important competence for nurses who seek autonomous practice, but also a differentiating characteristic of the professional to work in health services.

As a strategy to encourage entrepreneurship in undergraduate Nursing, the Junior Companies (JCs) can be mentioned. These are educational non-profit associations, formed and managed only by undergraduate students, who provide services under the guidance of professors. In their structure, they are identical to real companies, with principles of corporate governance and own regulation. Because they have an educational purpose, they are known for quality services, but with lower prices^(^ 29 ^-^ 30 ^)^.

The central objective of the JCs is to consolidate and reinforce learning, while contributing to public and private organizations. In addition to teamwork, the students develop other attributes, such as: leadership, communication, negotiation, and development of products and projects^(^ 30 ^)^. In Brazil, there are nearly 900 Junior Companies and 22,000 junior entrepreneurs, who carry out approximately 17,000 projects a year. However, there are only three federated JCs in the area of Nursing^(^ 31 ^)^.

In a Junior Nursing Company, the undergraduate students, under the supervision of professors, can apply their knowledge in the development of projects for public or private organizations such as schools, daycare centers, hospitals and the community in general, contributing to the realization of safe care for the population and for the improvement of permanent education for health and Nursing professionals^(^ 29 ^)^.

Internationally, other learning strategies and methodologies have been incorporated into Nursing training to improve entrepreneurship. The entrepreneurial education model in Nursing developed in South Korea suggests the inclusion of theoretical and practical content on entrepreneurship in the curriculum of the Nursing course, lectures with entrepreneur nurses, and development of internship programs linked to clinics and/or professionals with autonomous practice. It also points out the importance of complementary short courses on financial accounting, marketing, leadership, organizational culture, and development of startups^(^ 32 ^)^.

In Ireland, a number of researchers also highlight the potential of games and simulations for developing entrepreneurial skills, such as decision-making, risk management, problem solving, communication, and teamwork. These strategies allow for an innovative and contextualized learning environment for the students, based on the creation of real-world scenarios and situations^(^ 33 ^)^.

The development of university extension projects is an alternative for learning Nursing care as a socially entrepreneurial practice. As an example, a project developed by Brazilian Nursing professors and students in an Association of Recyclable Materials can be noted, based on care interventions that involve conversation circles, health education workshops, and social gatherings on special dates. The insertion of the university in the community constitutes an entrepreneurial strategy and proposes changes in a creative way and with the active participation of the workers for a more effective and resolving approach to health and well-being issues^(^ 6 ^)^.

Another successful experience is the Health Care Innovation Program developed in the United States. The objective of this program is to develop knowledge, skills and attitudes of future nurses to identify problems, generate scalable creative ideas and, subsequently, develop these innovations to meet the health care needs of patients, families and communities. For this, the main strategy used is the development of a business or innovation plan, in which the student must describe the clinical need for innovation, the target market, the end users and/or buyers of the innovation, competition in the market, and the implementation strategy^(^ 17 ^)^.

The results of this research contribute to the identification of the entrepreneurial tendency of Nursing students throughout the undergraduate course, by means of a multicenter study. In this sense, the need to invest in entrepreneurial training of nurses is pointed out in order to meet the social and health care demands, as well as to search for innovations and technologies in the face of changes in the labor market in the contemporary context. It is hoped that the findings presented can contribute to the practice of university professors and managers, encouraging discussions about curricular changes and strategies aimed at developing skills for entrepreneurship in Nursing.

This research has some limitations. One of them was the choice of convenience sampling, which was considered the best strategy for optimizing data collection in the different study scenarios. Another limitation refers to the cross-sectional design, in which reverse causality cannot be ruled out. In addition, any differences in the way in which the theme of entrepreneurship is addressed in the different Nursing courses in which data were collected were not considered. Such weaknesses indicate possibilities to be explored in future studies aiming to deepen the investigated theme.

## Conclusion

Beginning and concluding students of the undergraduate Nursing course showed a low entrepreneurial tendency. However, the concluding students were above the mean in the *Impulse and Determination* dimension. The study also showed that there is dissonance in the students’ perception between the importance of entrepreneurship in Nursing and its approach as a content throughout university education. Therefore, the importance of the investment of universities in the development of an entrepreneurial culture in higher education in Nursing is highlighted.
